# Our Publishing House

**Published:** 1878-01-20

**Authors:** 


OUR PUBLISHING HOUSE.—We have engaged to publish THE RECORD, the present year, J. H. Seals, editor of that popular literary paper, the *Sunny South*. After the first number, which will be a little delayed, we have assurance that our journal will be punctually furnished on the day of publication, (the 20th of each month.)

